# Preparative Isolation and Purification of Four Compounds from *Cistanches deserticola* Y.C. Ma by High-Speed Counter-Current Chromatography

**DOI:** 10.3390/molecules17078276

**Published:** 2012-07-10

**Authors:** Lifeng Han, Lina Ji, Mavis Boakye-Yiadom, Wei Li, Xinbo Song, Xiumei Gao

**Affiliations:** 1 Tianjin State Key Laboratory of Modern Chinese Medicine, Tianjin University of Traditional Chinese Medicine, 312 Anshanxi Road, Tianjin 300193, China; 2 Tianjin Key Laboratory of TCM Chemistry and Analysis, Tianjin University of Traditional Chinese Medicine, 312 Anshanxi Road, Tianjin 300193, China; 3 Inner Mongolia Mandela Sand Industry Development Co., Ltd., Left Banner of Alashan, Inner Mongolia 750300, China

**Keywords:** high-speed counter-current chromatography, *Cistanches deserticola* Y.C. Ma, phenylethanoid glycosides, acteoside, isoacteoside, syringalide A 3'-*α*-L-rhamnopyranoside, 2'-acetylacteoside

## Abstract

Following a constituent enrichment step on a silica gel column, four phenyl-ethanoid glycosides were successfully isolated from *Cistanches deserticola* and purified by preparative high-speed counter-current chromatography (HSCCC) with a two-phase solvent system composed of ethyl acetate-*n*-butanol-ethanol-water (40:6:6:50, *v/v/v/v*). A total of 30.9 mg acteoside, 13.0 mg isoacteoside, 12.5 mg syringalide A 3'-*α*-L-rhamnopyranoside and 7.2 mg 2'-acetylacteoside with purity of higher than 95%, as determined by HPLC-ELSD, were obtained in one-step separation from 297 mg of *Cistanche deserticola* extract, respectively. Their structures were identified by HR-MS, ^1^H-NMR and ^13^C-NMR.

## 1. Introduction

*Cistanches deserticola* Y.C. Ma, a species of *Cistanches* which belongs to the *Orobanchaceae* family, is a well-known Traditional Chinese Medicine for the treatment of kidney deficiency, female infertility, morbid leucorrhea, neurataxia and senile constipation [1]. It is a parasitic plant which is widely distributed in the northwest of China. So far, a number of compounds, including phenylethanoid glycosides (PhGs), iridoids and lignans have been isolated from this species [2]. Some of the PhGs have been reported to possess antioxidative, hepatoprotective and neuroprotective activities [3,4,5,6]. However, the isolation and purification procedure of PhGs is difficult and time-consuming. Moreover, the classical multiple chromatographic methods not only use a large amount of organic solvents but also give a lower sample recovery. High-speed counter-current chromatography (HSCCC), a support free liquid-liquid partition chromatographic technique, offers excellent sample recovery compared to some conventional methods, and has been widely used for separation and purification of various natural products [7,8,9]. Although several PhGs have been purified from *Cistanches* genus and others by HSCCC [10,11,12,13,14], no paper has reported the purification of acteoside, isoacteoside, syringalide A 3'-*α*-L-rhamnopyranoside and 2'-acetylacteoside in one biphasic system.

**Figure 1:**
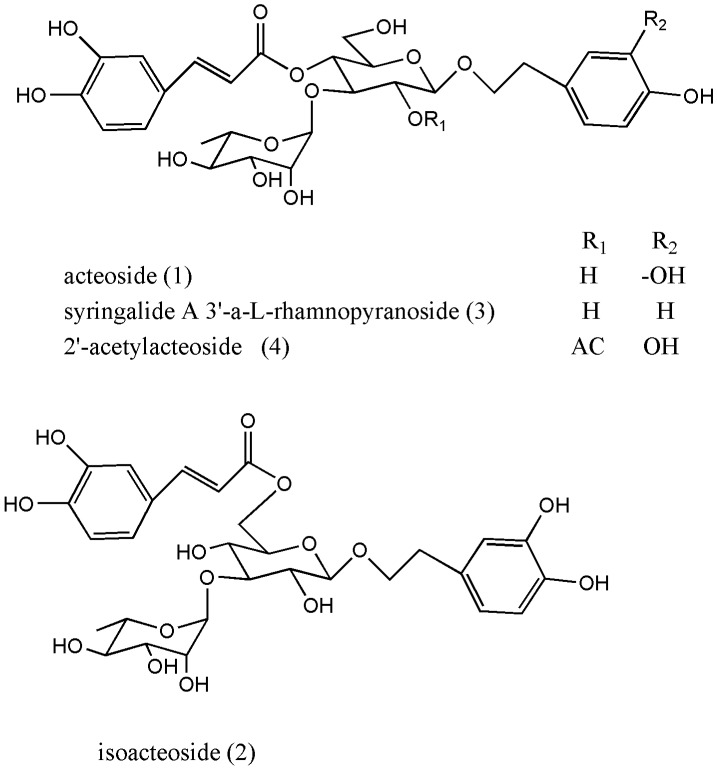
The structures of four isolated compounds.

In this paper, we report a simple method for the separation and purification of four PhGs from *C. deserticola.* Finally, 30.9 mg of acteoside, 13.0 mg of isoacteoside, 12.5 mg of syringalide A 3'-*α*-L-rhamnopyranoside and 7.2 mg of 2'-acetylacteoside were obtained in a one-step separation from 297 mg of fraction with purities of 99%, 95%, 99% and 98%, respectively. Their structures were characterized on the basis of ^1^H- and ^13^C-NMR spectral data and HR-MS spectra. The structures of the four PhGs identified in this investigation are shown in Figure 1.

## 2. Results and Discussion

### 2.1. HPLC Analysis of the Crude Extract

**Figure 2:**
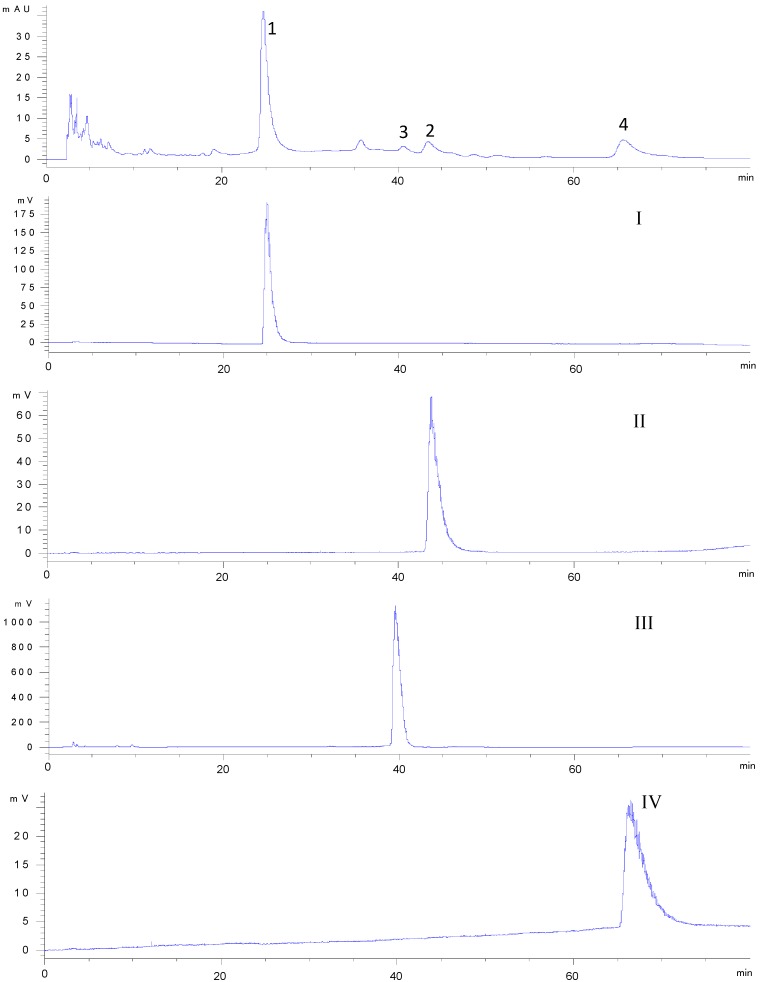
HPLC-UV/ELSD chromatograms of enriched crude extract and the four fractions.

The constituent enriched fraction of *n*-butanolic extract from *C. deserticola* was analyzed by HPLC-UV. The column used was an Accurasil C18 (250 mm × 4.6 mm, i.d. 5 μm) (Thermo-Fisher Scientific Instruments, city, state abbrev, USA), the mobile phase was methanol-water (30:70, *v/v*). The flow rate was 1 mL/min. Detector wavelength was set at 254 nm. The HPLC chromatogram is shown in Figure 2. Peaks 1 to 4 correspond to acteoside (**1**), isoacteoside (**2**), syringalide A 3'-*α*-L-rhamnopyranoside (**3**) and 2'-acetylacteoside (**4**), respectively.

### 2.2. Selection of Two-Phase Solvent System

Successful separation by HSCCC largely depends upon the selection of a suitable two-phase solvent system, the two phase solvent system was selected according to the partition coefficient values (*K*) of each target component and an optimum range of *K* should be from 0.5 to 2.

**Figure 3:**
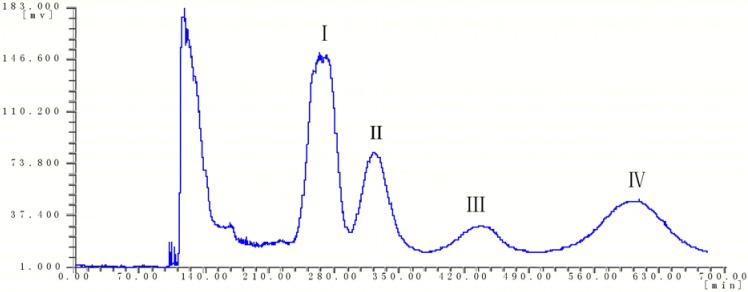
Chromatogram of HSCCC for separation of *C. deserticola*.

In the experiment, the *K* values for target components of several two phase solvent system are dshown in Table 1 (although a little higher for peak 4, the results were shown to be acceptable). Among them, ethyl acetate–*n*–butanol–ethanol–water (40:6:6:50, *v/v/v/v*) gave suitable partition coefficients for the target compounds. Eventually, the solvent system composed of ethyl acetate–*n*–butanol– ethanol–water (40:6:6:50, *v/v/v/v*) was used to isolate and purify four compounds of *C. deserticola*, as shown in Figure 3. As shown in Figure 2, the HPLC analysis of each HSCCC fraction revealed that four pure PhGs (acteoside, 99%; isoacteoside, 95%; syringalide A 3′-*α*-L-rhamnopyranoside 99% and 2′-acetylacteoside 98%) could be obtained from the crude fraction.

**Table 1:** *K* values of the compounds in different solvents system with different radios.

Solvent systems	K1	K2	K3	K4
ethyl acetate– *n*-butanol–ethanol–water (35:7:6:50, v/v/v/v)	1.78	2.96	3.00	5.37
ethyl acetate– *n*-butanol–ethanol–water (40:6:7:45, v/v/v/v)	1.68	2.42	3.01	4.3
ethyl acetate– *n*-butanol–ethanol–water (40:6:5:55, v/v/v/v)	1.48	2.94	2.33	3.49
ethyl acetate– *n*-butanol–ethanol–water (45:5:6:50, v/v/v/v)	1.12	1.66	3.10	3.10
ethyl acetate– *n*-butanol–ethanol–water (40:6:6:50, v/v/v/v)	1.20	1.29	2.56	3.66

## 3. Experimental

### 3.1. Apparatus

The HSCCC equipment employed in the present study was a TBE-300B high-speed counter-current chromatography system (Shanghai Tauto Biotech Co., Shanghai, China) with three preparative multilayer coil separation columns connected in series (diameter of tube = 2.6 mm, total volume = 300 mL) and a 20 mL sample loop. The revolution radius or the distance between the holder axis and central axis of the centrifuge (*R*) was 5 cm, and the *β* value varied from 0.5 at the internal terminal to 0.8 at the external terminal (*β *= *r*/*R*, where *r* is the distance from the coil to the holder shaft). An HX 105 constant-temperature circulation implement (Beijing Changliu Lab Instrument Company, Beijing, China) was used to control temperature in the experiment. The HSCCC system was equipped with a model TBP-5002 middle pressure constant-flow pump, a model TBP-2000 UV detector operating at 254 nm, and a model V4.0 workstation (Jinda Bio-chemistry Instrument Company, Shanghai, China).

The high-performance liquid chromatography (HPLC) equipment used was an Agilent 1200 system which consists of a G1312A binary pump, a G1329A autosampler, a G1314A variable wavelength detector (VWD), a G1316A thermostatted column compartment, a G1379B degasser and Agilent LC workstation. An alltech 3300 ELSD was used for detection of the purities. Agilent 6520 Q-TOF and Bruker AVANCE III 500-NMR spectrometers were used for compounds’ structural identification. ^1^H and ^13^C-NMR spectra (at 500 and 125 MHz, respectively) were measured at room temperature (22 °C/295.1 K).

### 3.2. Reagents and Materials

Ethyl acetate, *n*-butanol and ethanol were analytical grade and methanol was HPLC grade. All the solvents were purchased from Tianjin Concord Technology Company (Tianjin, China). Milli-Q water (18.2 MΩ) (Millipore, Bedford, MA, USA) was used for all solutions and dilutions. The rhizome of *C. deserticola* was collected from the Inner-Mongolia Province, People’s Republic of China, in June 2009. The plant was identified by Prof. Lijuan Zhang, and a voucher specimen (No. 20091001) was deposited in our laboratory.

### 3.3. Preparation of Crude Sample

Powdered air dried fleshy stems of *C. deserticola* (1 Kg) were refluxed twice with aqueous ethanol (8 L, 60% *v/v*) for 2 h each time. The extract was evaporated under reduced pressure and at a temperature of 60 °C until total evaporation of the ethanol. The residue was suspended in water and extracted successively three times with chloroform, ethyl acetate and *n*-butanol, affording 5.16 g of *n*-butanolic extract after evaporation to dryness under reduced pressure. The *n*-butanolic extract was then separated on a silica gel column (200–300 mesh) using progressive gradient elution (CHCl_3_–MeOH, 10:1→1:1) to give eight fractions. Fraction 6 (297 mg) was used for further HSCCC isolation and separation.

### 3.4. Selection of the Two-Phase-Solvent Systems

The two-phase solvent systems were selected according to the partition coefficient (*K*) of the target components in the fraction of *C. deserticola*. The *K* values were determined by LC analysis as follows: Suitable amount of crude extract powder (fraction 6) was dissolved in the lower phase of the solvent system and analyzed by HPLC. The areas of the peaks were recorded as *A*1. Then equal volume of the upper phase was added to the solution and mixed thoroughly to reach partition equilibrium. The lower phase was then analyzed by HPLC again. The latter peak areas were recorded as *A*2. The *K*-values were calculated according to the following equation: *K *= (*A*1 − *A*2)/*A*2 [15,16].

### 3.5. Preparation of Two-Phase-Solvent System and Sample Solution

In the present study, the two-phase solvent system composed of ethyl acetate–*n*-butanol–ethanol–water (40:6:6:50, *v/v/v/v*) was used for HSCCC separation. Each solvent was added to a separatory funnel and thoroughly equilibrated at room temperature. The upper phase and the lower phase were separated and degassed by sonication for 30 min shortly before use. The sample solution was prepared by dissolving 297 mg of Fraction 6 in 15 mL of the lower phase of ethyl acetate–*n*-butanol–ethanol–water (40:6:6:50, *v/v/v/v*).

### 3.6. HSCCC Separation Procedure

HSCCC was performed as follows. The multilayer coiled column was first entirely filled with the upper organic stationary phase. The lower aqueous mobile phase was then pumped into the head end of the column at a flow-rate of 1.5 mL/min, and at the same time, the HSCCC apparatus was run at a revolution speed of 900 rpm. After hydrodynamic equilibrium was established, as indicated by a clear mobile phase eluting at the tail outlet ( about two hours later ), 15 mL sample solution containing 297 mg of the crude extract (fraction 6) was injected through the injection valve. The effluent of the column was continuously monitored under 254 nm. Four peak fractions were collected according to the chromatogram and then evaporated under reduced pressure. The temperature of the apparatus was set at 25 °C.

### 3.7. HPLC Analysis and Identification of HSCCC Peak Fractions

The peak fractions from HSCCC were analyzed by HPLC-ELSD. The column used was an Accurasil C18 (250 mm × 4.6 mm, i.d. 5 μm), the mobile phase was methanol–water (30:70, *v/v*). The flow rate was 1 mL/min. ELSD was operated under the following conditions: Temp 45 °C, gas 1.6 L/min. Structural identification of the four HSCCC peak fractions was carried out by HR-ESI-MS, ^1^H and ^13^C-NMR spectroscopy.

### 3.8. The Structural Identification

Fraction **I**: HR-ESI-MS observed at *m*/*z* 623.2001 (M−H)^−^, calcd for C_29_H_35_O_15_, 623.1981. ^1^H-NMR (500 MHz, CD_3_OD) *δ* ppm: 1.09 (3H, d, *J *= 6 Hz, CH_3_ of rhamnose), 2.79 (2H, t, *J *= 7.5 Hz, Ar-CH_2_-), 4.37 (1H, d, *J *= 8 Hz, H-1 of glucose), 5.18 (1H, d, *J *= 1 Hz, H-1 of rhamnose), 6.27 (1H, d, *J* = 15.5 Hz, Ar-CH=CH-), 7.59 (1H, d, *J *= 15.5 Hz, Ar-CH=CH-), 6.5–7.1 (6H, aromatic H).^ 13^C-NMR (125 MHz, CD3OD) *δ* ppm: 131.5 (C-1), 117.2 (C-2), 146.2 (C-3), 144.7 (C-4), 116.4 (C-5), 121.3 (C-6), 72.4 (C-α), 36.6 (C-β), 127.7 (Caf-1), 115.3 (Caf-2), 146.9 (Caf-3), 149.8 (Caf-4), 116.6 (Caf-5), 123.2 (Caf-6), 168.3 (Caf-α), 114.8 (Caf-β), 148.1 (Caf-γ), 104.3 (G-1), 76.1 (Glc-2), 81.7 (Glc-3), 70.5 (Glc-4), 76.3 (Glc-5), 62.4 (Glc-6), 103.1 (Rha-1), 72.3 (Rha-2), 72.1 (Rha-3), 73.9 (Rha-4), 70.7 (Rha-5), 18.5 (Rha-6). Compared with the data given in literature [17], fraction I corresponded to acteoside.

Fraction **II**: HR-ESI-MS observed at *m*/*z* 623.1987 (M−H)^−^, calcd for C_29_H_35_O_15_, 623.1981. ^1^H-NMR (500 MHz, CD_3_OD) *δ* ppm: 1.25 (3H, d, *J *= 6.5 Hz, CH_3_ of rhamnose), 2.78 (2H, t, *J* = 7 Hz, Ar-CH_2_-), 4.33 (1H, d, *J *= 8 Hz, H-1 of glucose), 5.17 (1H, d, *J *= 1 Hz, H-1 of rhamnose), 6.28 (1H, d, *J *= 15.5 Hz, Ar-CH=CH-), 7.56 (1H, d, *J *= 15.5 Hz, Ar-CH=CH-), 6.5–7.0 (6H, aromatic H). ^13^C-NMR (125 MHz, CD_3_OD) *δ* ppm: 131.5 (C-1), 117.2 (C-2), 146.2 (C-3), 144.7 (C-4), 116.5 (C-5), 121.3 (C-6), 72.4 (C-α), 36.7 (C-β), 127.8 (Caf-1), 115.2 (Caf-2), 146.8 (Caf-3), 149.7 (Caf-4), 116.6 (Caf-5), 123.2 (Caf-6), 169.2 (Caf-α), 115.0 (Caf-β), 147.3 (Caf-γ), 104.5 (G-1), 75.5 (Glc-2), 84.2 (Glc-3), 70.1 (Glc-4), 75.7 (Glc-5), 64.7 (Glc-6), 102.8 (Rha-1), 72.4 (Rha-2), 72.4 (Rha-3), 74.1 (Rha-4), 70.5 (Rha-5), 17.9 (Rha-6). The ^1^H-NMR and ^13^C-NMR spectral data were in agreement with those of isoacteoside as reported in the literature [18].

Fraction **III**: HR-ESI-MS observed at *m*/*z* 607.2032 (M−H)^−^, calcd for C_29_H_35_O_14_, 607.2032. ^1^H-NMR (500 MHz, CD_3_OD) *δ* ppm: 1.09 (3H, d, *J *= 6 Hz, CH_3_ of rhamnose), 2.84 (2H, t, *J* = 7.5 Hz, Ar-CH_2_-), 4.37 (1H, d, *J *= 8 Hz, H-1 of glucose), 5.19 (1H, d, *J *= 1.5 Hz, H-1 of rhamnose), 6.27 (1H, d, *J *= 16 Hz, Ar-CH=CH-), 7.59 (1H, d, *J *= 16 Hz, Ar-CH=CH-), 6.6–7.1 (7H, aromatic H). ^13^C-NMR (125 MHz, CD_3_OD) *δ* ppm: 130.8 (C-1), 116.2 (C-2), 130.9 (C-3), 156.8 (C-4), 130.8 (C-5), 116.2 (C-6), 72.4 (C-α), 36.4 (C-β), 127.8 (Caf-1), 114.8 (Caf-2), 149.8 (Caf-3), 146.9 (Caf-4), 116.6 (Caf-5), 123.2 (Caf-6), 168.3 (Caf-α), 115.4 (Caf-β), 148.0 (Caf-γ), 104.3 (G-1), 76.3 (Glc-2), 81.7 (Glc-3), 70.4 (Glc-4), 76.1 (Glc-5), 62.5 (Glc-6), 103.0 (Rha-1), 72.3 (Rha-2), 72.2 (Rha-3), 73.9 (Rha-4), 70.7 (Rha-5), 18.4 (Rha-6). According to the literature [18], fraction III corresponded to syringalide A 3'-*α*-L-rhamnopyranoside.

Fraction **IV**: HR-ESI-MS observed at *m*/*z* 665.2100 (M−H)^−^, calcd for C_31_H_37_O_16_, 665.2087. ^1^H-NMR (500 MHz, CD_3_OD) *δ* ppm: 1.09 (3H, d, *J *= 6.5 Hz, CH_3_ of rhamnose), 2.00 (3H, s, OAc), 2.72 (2H, t, *J *= 7.5 Hz, Ar-CH_2_-), 4.55 (1H, d, *J *= 8 Hz, H-1 of glucose), 4.90 (1H, d, *J *= 1 Hz, H-1 of rhamnose), 6.29 (1H, d, *J *= 15.5 Hz, Ar-CH=CH-), 7.62 (1H, d, *J *= 15.5 Hz, Ar-CH=CH-), 6.5–7.0 (6H, aromatic H).^13^C-NMR (125 MHz, CD_3_OD) *δ* ppm: 131.9 (C-1), 117.3 (C-2), 146.1 (C-3), 144.7 (C-4), 116.4(C-5), 121.4 (C-6), 72.7 (C-α), 36.4 (C-β), 127.7 (Caf-1), 115.4 (Caf-2), 146.9 (Caf-3), 149.9 (Caf-4), 116.6 (Caf-5), 123.2 (Caf-6), 168.1 (Caf-α), 114.7 (Caf-β), 148.2 (Caf-γ), 101.8 (G-1), 75.3 (Glc-2), 80.3 (Glc-3), 70.8 (Glc-4), 76.2 (Glc-5), 62.3 (Glc-6), 103.3 (Rha-1), 72.0 (Rha-2), 71.8 (Rha-3), 73.7 (Rha-4), 70.8 (Rha-5), 18.5 (Rha-6), 171.5 (C=O), 20.9 (OAC). The ^1^H-NMR and ^13^C-NMR spectral data were in agreement with those of 2'-acetylacteoside [17].

## 4. Conclusions

An HSCCC method for the preparative separation and purification of acteoside, isoacteoside, syringalide A 3'-*α*-L-rhamnopyranoside and 2'-acetylacteoside from *Cistanches deserticola* Y.C. Ma was established. The present study indicates that HSCCC is a very powerful technique for the preparative separation and purification of bioactive components from plant materials. Also, the compounds can be isolated on a sufficiently large scale with high purities and may then be used as reference substances for chromatography or for bioactivity studies. The method is a feasible, economical, and efficient technique for rapid preparative isolation of complicated natural products.
